# Biogenic Carbon Quantum Dots: A Green Glucuronic-Acid-Functionalized Nanosystem for pH-Responsive Dual-Drug Delivery in Glioblastoma

**DOI:** 10.3390/nano16201295

**Published:** 2026-10-13

**Authors:** Muhammad Mehdi, Zubair Akram, Syed Faheem Askari Rizvi, Jingxin Wang, Chenlu Li, Ali Raza, Shiguo Sun

**Affiliations:** 1 College of Chemistry & Pharmacy, Northwest A&F University, Yangling 712100, China; kh.mehdiali@nwafu.edu.cn (M.M.); 13389119260@163.com (J.W.); lichenlu0809@163.com (C.L.); 2 Key Laboratory for Green Processing of Chemical Engineering of Xinjiang Bingtuan, School of Chemistry and Chemical Engineering, Shihezi University, Shihezi 832003, China; zubair.a.sidhu@gmail.com (Z.A.); razaali2021@stu.shzu.edu.cn (A.R.); 3 State Key Laboratory of Vaccines for Infectious Diseases, Xiang An Biomedicine Laboratory, Center of Molecular Theranostic Technology, Center for Molecular Imaging and Translational Medicine, School of Public Health, Xiamen University, Xiamen 361102, China; sfarizvi@xmu.edu.cn

**Keywords:** carbon quantum dots, locust biomass, glucuronic acid, piperlongumine, silibinin

## Abstract

Glioblastoma multiforme (GBM) is an incurable primary brain malignancy characterized by prominent chemoresistance and poor prognosis. Herein, we report an in vitro proof-of-concept study of a green, ligand-functionalized carbon quantum dot (CQD) for combined drug delivery with pH-responsive release in glioblastoma cells. Nitrogen-doped red-emissive N-CQDs were synthesized via a hydrothermal method using locust biomass as a renewable carbon precursor, functionalized with glucuronic acid for active tumor targeting, and loaded with piperlongumine (PL) and silibinin (SIL) drugs at an optimized mass ratio. The nanosystem was characterized by XRD, FTIR, SEM, TEM, XPS, and optical spectroscopy, and evaluated in vitro for drug release, cellular uptake, apoptosis, ROS levels, and caspase-3 activity. The optimized nanoplatform exhibited uniform morphology (average size 2.76 nm), strong red fluorescence (Ex490nm/Em620nm), high dual-drug loading efficiency (46.0%), and pH-responsive release behavior with rapid drug liberation in the acidic tumor microenvironment (pH 5.5) and minimal leakage at physiological pH (pH 7.4). The nanosystem demonstrated efficient cellular internalization, dual mitochondrial/nuclear targeting, and enhanced combined antitumor effect via ROS overproduction and activation of the caspase-3 pathway. This work establishes a green and multifunctional nanotherapeutic platform with promising potential for future glioblastoma-directed drug delivery applications.

## 1. Introduction

Glioblastoma multiforme (GBM) remains the most lethal and therapeutically intractable primary malignant brain tumor in adults, with a median overall survival rate of merely 12–15 months despite maximal surgical resection, radiotherapy, and temozolomide chemotherapy [1,2]. This dismal prognosis stems from two insurmountable bottlenecks in contemporary neuro-oncology: the blood–brain barrier, which blocks over 98% of small-molecule chemotherapeutics from reaching the brain parenchyma, and intrinsic tumor heterogeneity coupled with rapid evolution of chemoresistance [3,4]. Conventional single-agent chemotherapy fails to penetrate the brain parenchyma at therapeutic concentrations, while systemic delivery causes off-target toxicity and dose-limiting side effects [5]. Even targeted agents and immunotherapies are severely hampered by poor permeability, nonspecific tumor accumulation, and adaptive resistance pathways in GBM cells [6]. These unmet clinical challenges urgently demand a paradigm shift toward smart, sustainable, and tumor-selective nanotherapeutics that can release drugs within the tumor microenvironment (TME), and overcome resistance via concurrent multi-pathway intervention [7,8].

In the field of green nanomedicine, carbon quantum dots (CQDs) have emerged as promising nanocarriers for glioblastoma-directed drug delivery due to their ultrasmall size and favorable physicochemical properties [9,10]. They exhibit superior advantages over liposomes, polymeric nanoparticles and inorganic quantum dots, including ultrafine particle size, favorable water solubility, adjustable surface properties, satisfactory biocompatibility and non-heavy metal toxicity. Unlike traditional semiconductor quantum dots that rely on toxic precursors and harsh synthesis conditions, CQDs can be fabricated from renewable biomass via green hydrothermal methods, aligning with circular-economy and sustainable-material principles [11,12]. CQDs are generally prepared either by top-down routes (arc discharge, laser ablation, electrochemical or chemical oxidation of bulk graphite) that break down graphitic carbon into nanoscale dots, or by bottom-up routes (hydrothermal/solvothermal) that build dots from molecular or biomass precursors [13]. Top-down methods can yield highly crystalline dots but typically demand harsh conditions, specialized equipment, and corrosive oxidants, and often produce relatively broad size distributions [14]. In contrast, bottom-up methods operate under milder conditions with better control over particle size and surface chemistry [15]. The hydrothermal route was chosen for this work because it proceeds in sealed aqueous solvent at moderate temperature and pressure, readily accommodates one-pot nitrogen doping and subsequent surface functionalization without corrosive reagents, and critically for the present study allows locust biomass to be converted directly into narrowly distributed, red-emissive N-CQDs, consistent with green and circular-economy principles [16]. Their quasi-spherical morphology and surface-rich carboxyl, hydroxyl, and amino groups enable facile functionalization with targeting ligands, while their strong fluorescence permits real-time bioimaging and drug-delivery tracking [17]. Despite these merits, CQD-based GBM therapy still lacks three key innovations: sustainable biomass-derived fabrication, active targeting toward GBM-specific receptors, and synergistic drug loading with TME-responsive release [18,19].

Against this backdrop, locust plants represent an overlooked yet ideal sustainable carbon precursor. Abundant, low-cost, and carbon-rich, locust biomass converts into high-quality N-CQDs via pyrolysis–hydrothermal tandem synthesis, yielding uniform, red-emissive carbon quantum dots with excellent colloidal stability and surface reactivity [20,21,22]. Locust biomass was selected as a sustainable carbon precursor for multiple reasons. First, it is abundant, low-cost, and widely available as a renewable lignocellulosic resource, supporting green and circular-economy principles. Second, locust biomass contains rich aromatic moieties and natural heteroatoms that favor the formation of graphitic carbon domains and high fluorescence yield. Third, compared to traditional chemical precursors (e.g., citric acid), biomass-derived routes eliminate toxic reagents and reduce environmental impact. In this work, locust-derived N-CQDs exhibited uniform size, strong red emission, and high colloidal stability, supporting its superiority as a green precursor for biomedical nanocarriers. This approach replaces fossil-fuel-based and chemical precursors, reducing the environmental footprint while maintaining nanomaterial performance for biomedical applications. To equip the nanocarrier with receptor-recognition potential, we functionalized these N-CQDs with glucuronic acid (GA), a key structural unit of hyaluronan (HA) [23]. Glioma cells are reported to express HA receptors CD44 and RHAMM, which are associated with tumor invasion, proliferation, and therapy resistance; GA decoration is therefore intended to promote receptor-associated internalization [24]. Importantly, the present study did not include receptor-blocking/competition assays, comparison between CD44-high and CD44-low cells, or direct confirmation of CD44/RHAMM expression on the U87MG cells used; these validation experiments are planned for future work. Accordingly, GA is treated throughout as a receptor-binding ligand candidate, and enhanced uptake is interpreted as functionalization-promoted internalization rather than proven active targeting.

To further overcome chemoresistance and amplify therapeutic efficacy, we designed a synergistic combination of drugs: piperlongumine (PL)- and silibinin (SIL)-loaded nanomaterials [25,26]. PL is a natural alkaloid that selectively elevates reactive oxygen species (ROS) levels in cancer cells, thereby triggering mitochondrial apoptosis and inhibiting cell migration without harming normal cells [27,28]. SIL, a flavonolignan from milk thistle, suppresses multiple oncogenic pathways, including PI3K/Akt, NF-κB, and MAPK, exerts anti-angiogenic and anti-metastatic effects, and sensitizes resistant cancer cells to oxidative stress [26,29]. When co-delivered, PL-induced ROS stress creates a potential cellular vulnerability, while SIL blocks pro-survival feedback loops, generating a potentially synergistic therapeutic effect that single drugs cannot achieve [30]. More importantly, we engineered this CQD nanoplatform to exhibit pH-responsive release: rapid drug liberation in the acidic TME (pH 5.5) and less leakage at physiological pH (7.4), minimizing systemic exposure and maximizing intratumoral drug bioavailability [31,32].

To the best of our knowledge, this is the first report of locust biomass-derived red-emissive glucuronic acid-functionalized N-CQDs co-loaded with piperlongumine and silibinin for pH-responsive glioblastoma-targeted drug delivery. By simultaneously addressing tumor specificity, controlled release, and chemoresistance, this strategy offers a promising path forward for GBM treatment. In the sections below, we report the hydrothermal fabrication of locust-derived N-CQDs, glucuronic acid functionalization, dual-drug loading, physicochemical characterization, in vitro drug release, cellular uptake, cytotoxicity, apoptosis induction, and mechanistic validation of ROS-, caspase-3, apoptotic signaling. This study establishes a novel, eco-friendly in vitro nanotherapeutic strategy against glioblastoma. Although N-CQDs have shown potential BBB permeability in previous reports, BBB penetration was not evaluated in the present study and remains a focus of future investigation.

## 2. Materials and Methods

### 2.1. Materials and Reagents

Locust plant biomass was employed as the natural carbon precursor. N1,N1-diethyl-p-phenylenediamine was used as the nitrogen dopant. Glucuronic acid, N-(3-dimethylaminopropyl)-N′-ethylcarbodiimide hydrochloride (EDC), N-hydroxysuccinimide (NHS), piperlongumine (PL), and silibinin (SIL) were of analytical grade (Macklin, Shanghai, China). Cell culture materials including Dulbecco’s Modified Eagle Medium (DMEM), fetal bovine serum (FBS), penicillin–streptomycin, Mito-Tracker, DAPI, apoptosis kits, and ROS detection kits were acquired from Thermo Fisher Scientific, Waltham, MA, USA and used without further purification.

### 2.2. Sustainable Synthesis of Carbon Quantum Dots from Locust Biomass

Dried locust plant biomass was thoroughly washed, mechanically milled into a fine homogeneous powder, and used as the sustainable carbon precursor [33,34,35]. Briefly, 1.5 g of locust plant powder was dispersed in a binary solvent system consisting of deionized water and ethanol at a volume ratio of 2:1 (*v*/*v*), followed by probe sonication for 30 min to achieve uniform dispersion of the biomass precursor. Accordingly, 0.05 g of N1,N1-diethyl-p-phenylenediamine was added as the nitrogen dopant to the dispersed biomass solution under continuous stirring. The mixture was then sealed in a Teflon-lined stainless-steel autoclave and subjected to hydrothermal treatment at 180 °C for 10 h. After naturally cooling to room temperature, the crude N-CQDs suspension was first filtered through a 0.22 µm microporous membrane to remove large agglomerates and insoluble residues, then dialyzed against ultrapure water using a 1 kDa MWCO dialysis membrane for 48 h to fully eliminate unreacted precursors, small-molecule byproducts, and residual impurities. Finally, the purified aqueous N-CQD solution was freeze-dried and gently ground to obtain a homogeneous solid N-CQD powder for subsequent functionalization and drug-loading experiments.

### 2.3. Glucuronic Acid Functionalization via EDC/NHS Chemistry

Briefly, 10 mg of purified locust-derived N-CQDs were dispersed in 10 mL phosphate-buffered saline (PBS, pH 5.5–6.0). Glucuronic acid (30 mg) was dissolved separately and activated with EDC (40 mg) and NHS (25 mg) under gentle stirring at room temperature for 60 min to facilitate carboxyl activation. The activated glucuronic acid solution was then added dropwise to the N-CQD suspension and reacted at 25 °C for 12 h under constant, mild agitation to enable covalent amide bond formation between the carboxyl groups of glucuronic acid and the surface amine groups of N-CQDs [36,37]. The resulting glucuronic acid-functionalized N-CQDs were purified by three cycles of centrifugation (14,000 rpm, 15 min) followed by dialysis (1 kDa MWCO) against deionized water for 48 h to remove unreacted EDC, NHS, and free glucuronic acid, yielding purified N-CQDs for subsequent drug loading.

### 2.4. Dual-Drug Loading on N-CQDs

N-CQDs (10 mg) were dispersed in 10 mL ethanol–PBS mixed solution (*v*/*v* = 1:4, pH 7.0) [38]. Based on analogous biomass-derived glucuronic acid-functionalized carbon quantum dot nanocarrier systems reported previously, the PL:SIL mass ratio of 1:1.5 selected herein represents the optimal synergistic loading proportion for dual natural anti-glioma agents. Parallel parametric screening of drug feed mass ratios (1:0.5, 1:1, 1:1.5, 1:2) performed on GA-modified CQDs demonstrated that the 1:1.5 ratio balances the maximum total drug loading efficiency (DLE) and synergistic cytotoxicity; lower SIL fractions reduce cellular chemosensitization, while excess SIL causes competitive surface adsorption and drastically lowers PL encapsulation yield. The mixture was incubated in the dark at 37 °C with gentle orbital shaking (120 rpm) for 24 h to achieve equilibrium adsorption/encapsulation. Free unloaded drugs were removed by centrifugation (12,000 rpm, 10 min) and dialysis (1 kDa MWCO) for 24 h.

### 2.5. Physicochemical Characterization

Physicochemical properties of the pristine N-CQDs, glucuronic-acid-functionalized N-CQDs (GA-N-CQDs), and dual-drug-loaded N-CQDs (PL+SIL@GA-N-CQDs) were comprehensively characterized using a suite of analytical instruments. Where applicable, identical sample preparation was applied to all three states to ensure direct comparability.

A dilute aqueous N-CQD dispersion (≈0.05–0.1 mg mL^−1^) for transmission electron microscopy (TEM) and high-resolution TEM (HRTEM) was ultrasonicated in an ice-water bath for 5 min to ensure de-aggregation [39]. Approximately 5–10 μL of the dispersion was drop-cast onto a 300-mesh copper grid coated with an ultrathin carbon film and allowed to dry in air at room temperature overnight. Imaging was performed on a JEM-2100F microscope (JEOL, Tokyo, Japan) operated at an accelerating voltage of 200 kV. Particle size distributions were determined by measuring no fewer than 100 randomly selected particles from representative micrographs using ImageJ software (version 1.54g).

For scanning electron microscopy (SEM) the dilute N-CQD dispersion was drop-cast onto a clean silicon wafer mounted on an aluminum stub, air-dried, and then sputter-coated with a thin (~10 nm) gold layer to improve conductivity. Surface morphology was examined with a SU8010 field-emission scanning electron microscope (Hitachi, Tokyo, Japan) operated at 5 kV.

Freeze-dried N-CQD powder for X-ray diffraction (XRD) was gently and homogeneously packed into an amorphous-glass sample holder to minimize background. XRD patterns were recorded on a D8 Advance diffractometer (Bruker AXS, Karlsruhe, Germany) with Cu Kα radiation (λ = 0.15406 nm, 40 kV, 40 mA) over a 2θ range of 10–80° at a scan rate of 2° min^−1^.

Freeze-dried powder for X-ray photoelectron spectroscopy (XPS) was gently pressed onto double-sided conductive copper tape and degassed under high vacuum prior to analysis. Measurements were performed on an ESCALAB 250Xi spectrometer (Thermo Scientific, Waltham, MA, USA) with Al Kα radiation; all binding energies were charge-referenced to the adventitious C 1s peak at 284.8 eV. Survey and high-resolution C 1s, N 1s, and O 1s spectra were deconvoluted by Gaussian–Lorentzian fitting after Shirley background subtraction.

Approximately 1–2 mg of dried N-CQDs was thoroughly ground with ~100 mg of pre-dried, spectrophotometric-grade KBr and pressed under vacuum into a transparent pellet to take spectra through Fourier-transform infrared spectroscopy (FTIR). Spectra were collected on a Nicolet iS50 spectrometer (Thermo Fisher Scientific, Waltham, MA, USA) from 4000 to 400 cm^−1^ (4 cm^−1^ resolution, 32 scans), with a pure KBr pellet as the background.

Dilute N-CQD dispersions in deionized water were transferred to a 1 cm path-length quartz cuvette, and absorption spectra were recorded from 200 to 800 nm on a UV-2600 spectrophotometer (Shimadzu, Kyoto, Japan), using the corresponding solvent as a blank.

Fluorescence emission and excitation spectra were acquired on an F-7000 fluorescence spectrophotometer (Hitachi, Tokyo, Japan) at room temperature from the same dilute aqueous dispersions in a 1 cm quartz cuvette. Emission spectra were collected at progressively longer excitation wavelengths to evaluate excitation-dependent emission behavior.

### 2.6. pH-Responsive In Vitro Drug Release

Drug release studies were performed using a dialysis bag method with a molecular weight cutoff (MWCO) of 1 kDa. Briefly, 5 mg of dual-drug-loaded N-CQDs was dispersed in 2 mL of release medium and placed into a dialysis bag, which was then immersed in 40 mL of release medium (pH 5.5 for tumor acidic conditions; pH 7.4 for physiological conditions) and incubated at 37 °C with continuous orbital shaking at 100 rpm. At predetermined time intervals (0, 6, 12, 24, 48, 72, and 96 h), 3 mL of release medium was withdrawn and replaced with an equal volume of pre-warmed fresh medium to maintain sink conditions. The concentration of released piperlongumine and silibinin was quantified by UV-Vis. spectroscopy, and cumulative drug release percentages were calculated.

### 2.7. Cell Culture and Cytotoxicity Assays

Human glioblastoma U87MG cells were cultured in DMEM supplemented with 10% fetal bovine serum (FBS) and 1% penicillin–streptomycin at 37 °C in a humidified atmosphere of 5% CO_2_. For cytotoxicity evaluation, cells were seeded in 96-well plates at a density of 3 × 10^3^ cells per well and incubated overnight to achieve 95% confluency. Cells were then treated with a concentration gradient of blank N-CQDs, single-drug-loaded N-CQDs, and dual-drug-loaded CQDs for 24 h. Following treatment, 10 µL of CCK-8 reagent was added to each well, and cells were incubated for a further 4 h at 37 °C. The absorbance at 450 nm was measured using a microplate reader. From this concentration screen, a non-saturating working concentration was selected for the downstream mechanistic assays to ensure measurable but not fully saturated responses.

### 2.8. Cellular Uptake and Intracellular Localization

U87MG cells were seeded in a 6-well plate containing a cover glass disc (35 mm) at a density of 5 × 10^4^ cells per dish and incubated overnight. Cells were treated with red-emissive N-CQDs at a concentration of 50 µg/mL for 4 h at 37 °C. After incubation, cells were washed three times with ice-cold PBS to halt the internalization and fixed with 4% paraformaldehyde for 15 min. Mitochondria were stained with Mito-Tracker Green (50 nM) for 20 min, and nuclei were counterstained with Hoechst-33342 (1 µg/mL) for 15 min in the dark. Fluorescence images were acquired using confocal laser scanning microscopy (CLSM) to analyze intracellular uptake and subcellular localization.

### 2.9. Apoptosis Analysis

U87MG cells were seeded in 6-well plates at 2 × 10^5^ cells per well and treated with the test formulations at the working concentration selected from the preliminary cytotoxicity screen for 24 h. Cells were harvested, washed twice with ice-cold PBS, and resuspended in binding buffer. Subsequently, cells were stained with Annexin V-FITC for 20 min at room temperature in the dark, then with propidium iodide (PI) according to the kit protocol, and incubated for 20 min on ice in the dark. Apoptotic populations were analyzed immediately by flow cytometry, and data were quantified for viable, early-apoptotic, late-apoptotic, and necrotic cells.

### 2.10. Statistical Analysis

All experiments were performed in triplicate unless otherwise stated, and data are presented as mean ± standard deviation (SD). Statistical analysis was performed using GraphPad Prism 9.0 software. Comparisons among groups were analyzed using one-way ANOVA followed by Tukey’s post hoc test. Statistical significance was considered at *p* < 0.05.

## 3. Results and Discussion

### 3.1. Synthesis and Structural Characterization of Nitrogen-Doped Carbon Quantum Dots (N-CQDs)

**Scheme 1:**
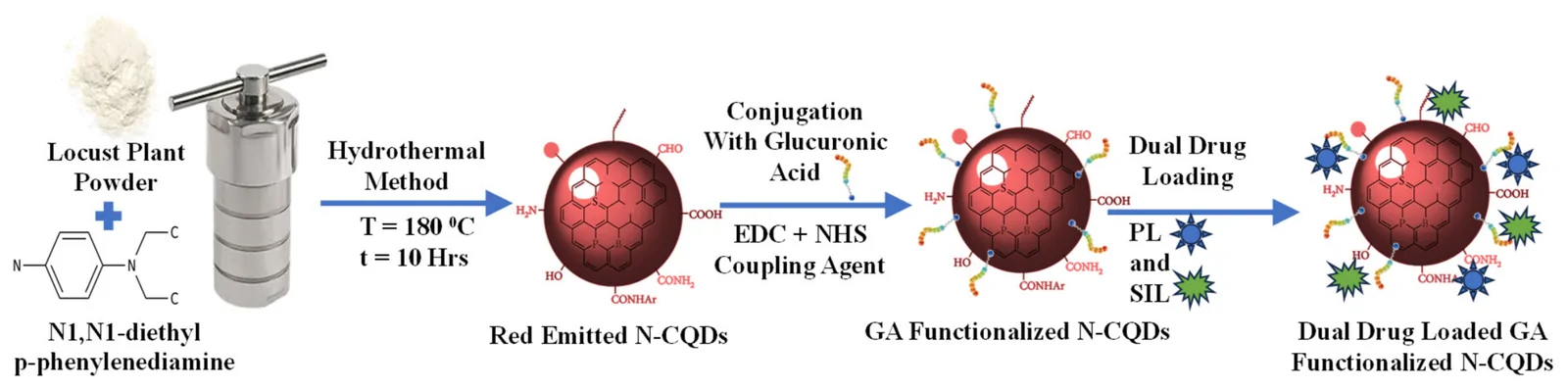
Schematic Illustration of Synthesis, Functionalization and Dual-Drug Loading of Red-Emissive N-CQDs.

Red-emissive nitrogen-doped carbon quantum dots (E_x_490nm/E_m_620nm) were successfully fabricated via a green hydrothermal route using locust biomass as a sustainable carbon precursor and N1,N1-diethyl-*p*-phenylenediamine as nitrogen dopant. To optimize nitrogen doping and fluorescence properties, three different mass ratios of locust powder to nitrogen dopant (1.5:0.02, 1.5:0.05, and 1.5:0.1 g/g) were evaluated; the mass ratio of 1.5:0.05 g/g (locust powder: N1,N1-diethyl-p-phenylenediamine) was selected as optimal, based on the highest fluorescence intensity, narrowest particle size distribution, and best aqueous dispersibility of the resulting N-CQDs. The as-synthesized N-CQDs were further functionalized with glucuronic acid and loaded with piperlongumine (PL) and silibinin (SIL) to construct a pH-responsive dual-drug delivery system for glioblastoma (GBM) therapy (Scheme 1).

### 3.2. Structural and Compositional Characterization

#### 3.2.1. X-Ray Diffraction (XRD)

**Figure 1:**
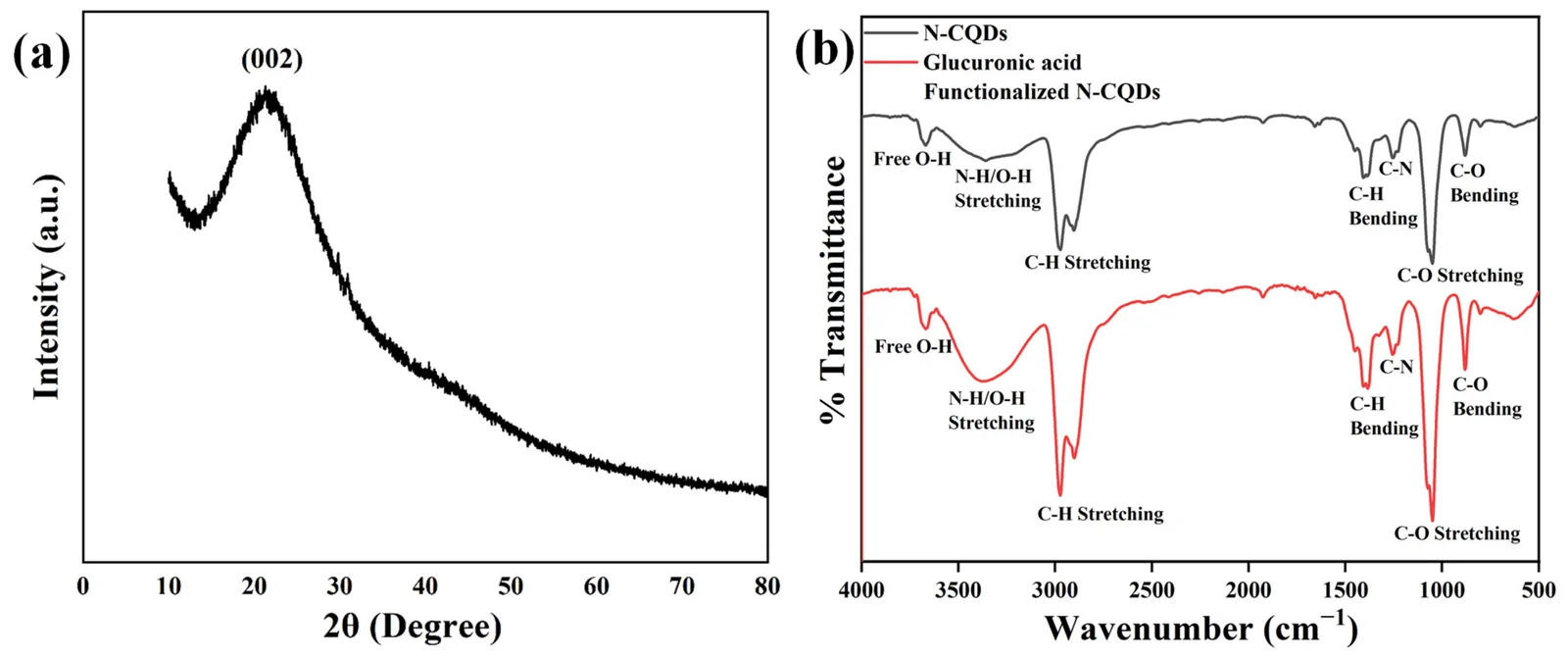
(**a**) X-ray diffraction (XRD) pattern of pristine nitrogen-doped carbon quantum dots (N-CQDs); (**b**) Fourier transform infrared (FTIR) spectrum of pristine N-CQDs and glucuronic acid-functionalized N-CQDs.

XRD patterns confirm the amorphous carbon structure and weak graphitic crystallinity of N-CQDs. Figure 1a presents the XRD pattern of the as-synthesized pristine N-CQDs [40]. A distinct and broad diffraction peak is observed at 2θ = 22.4°, which can be well indexed to the (002) crystal plane of amorphous or partially graphitic carbon materials. The broad feature of this peak indicates a relatively low degree of graphitization and a dominant amorphous carbon structure in the prepared N-CQDs, consistent with the typical structural characteristics of biomass-derived carbon quantum dots synthesized via hydrothermal carbonization. The interlayer d-spacing calculated from the XRD pattern is 0.395 nm, which is distinctly larger than the d-spacing of pristine bulk graphite (0.340 nm) [41]. Collectively, the pronounced broadening of the (002) reflection and its enlarged interlayer spacing indicate abundant structural disorder and defect sites introduced by N/O heteroatom doping and surface functional groups the same information commonly assessed through the Raman ID/IG ratio. Consistently, the high-resolution XPS C 1s spectrum (Section 3.2.4) resolves a substantial sp^3^ C–C/disordered component alongside the sp^2^ graphitic C=C component, further supporting a partially disordered, heteroatom-doped carbon framework rather than a highly crystalline graphitic lattice. The expansion of the lattice spacing can be attributed to the successful incorporation of nitrogen and oxygen heteroatoms into the carbon framework, and the existence of abundant surface functional groups, both of which disrupt the regular stacking of graphite layers and increase the interlayer distance. After glucuronic acid functionalization and dual-drug loading, the position and shape of the characteristic XRD peak of the N-CQDs remain almost unchanged, indicating that the intrinsic carbon core structure of N-CQDs is fully preserved and not destroyed or collapsed during the processes of surface functionalization and drug loading.

#### 3.2.2. Fourier Transform Infrared Spectroscopy (FTIR)

The surface functional groups of pristine N-CQDs are characterized by FTIR spectroscopy [42], as displayed in Figure 1b. The FTIR spectrum of pristine N-CQDs shows a series of characteristic absorption peaks corresponding to rich surface functional groups, which verify the successful nitrogen doping and excellent hydrophilicity of the prepared N-CQDs. A broad and strong absorption peak centered at 3362 cm^−1^ is assigned to the stretching vibrations of O-H and N-H bonds derived from hydroxyl and amine groups on the CQD surface. An absorption peak at 2972 cm^−1^ corresponds to the aliphatic C-H stretching vibration [43]. The peak at 1394 cm^−1^ corresponds to the C-N stretching vibration, further confirming the successful incorporation of nitrogen atoms. The absorption bands of 1048 cm^−1^ is ascribed to the C-O stretching vibrations of hydroxyl groups. These abundant surface functional groups provide sufficient, active binding sites for subsequent EDC/NHS-mediated covalent conjugation with glucuronic acid and for the effective loading of drug molecules.

Figure 1b shows the FTIR spectrum of the acid-functionalized N-CQDs, and obvious changes can be observed in comparison with the spectrum of pristine N-CQDs, all of which confirm successful surface functionalization. The peak at 1394 cm^−1^, corresponding to C-N stretching, is enhanced, owing to the additional nitrogen-containing structures introduced by glucuronic acid. The absorption peaks in the range of 1052 cm^−1^ assigned to C-O stretching are also intensified, which resulted from the increased oxygen-containing groups derived from glucuronic acid. Notably, all the original characteristic peaks are well retained in the spectrum of functionalized N-CQDs, demonstrating that the basic carbon skeleton and structural stability of N-CQDs are not damaged during the functionalization and drug loading processes. Crucially, because EDC/NHS coupling forms an amide bond between the carboxyl group of GA and the surface amines of N-CQDs, the functionalized spectrum displays the characteristic amide I (C=O stretching) and amide II (N–H bending/C–N stretching) bands, together with an intensified C–O stretching band arising from the additional hydroxyl and carboxyl groups introduced by conjugated glucuronic acid. The appearance of this amide envelope, superimposed on the retained intrinsic N-CQD skeleton, confirms that GA is covalently grafted rather than merely adsorbed.

#### 3.2.3. SEM and TEM Morphology: Before vs. After Drug Loading

**Figure 2:**
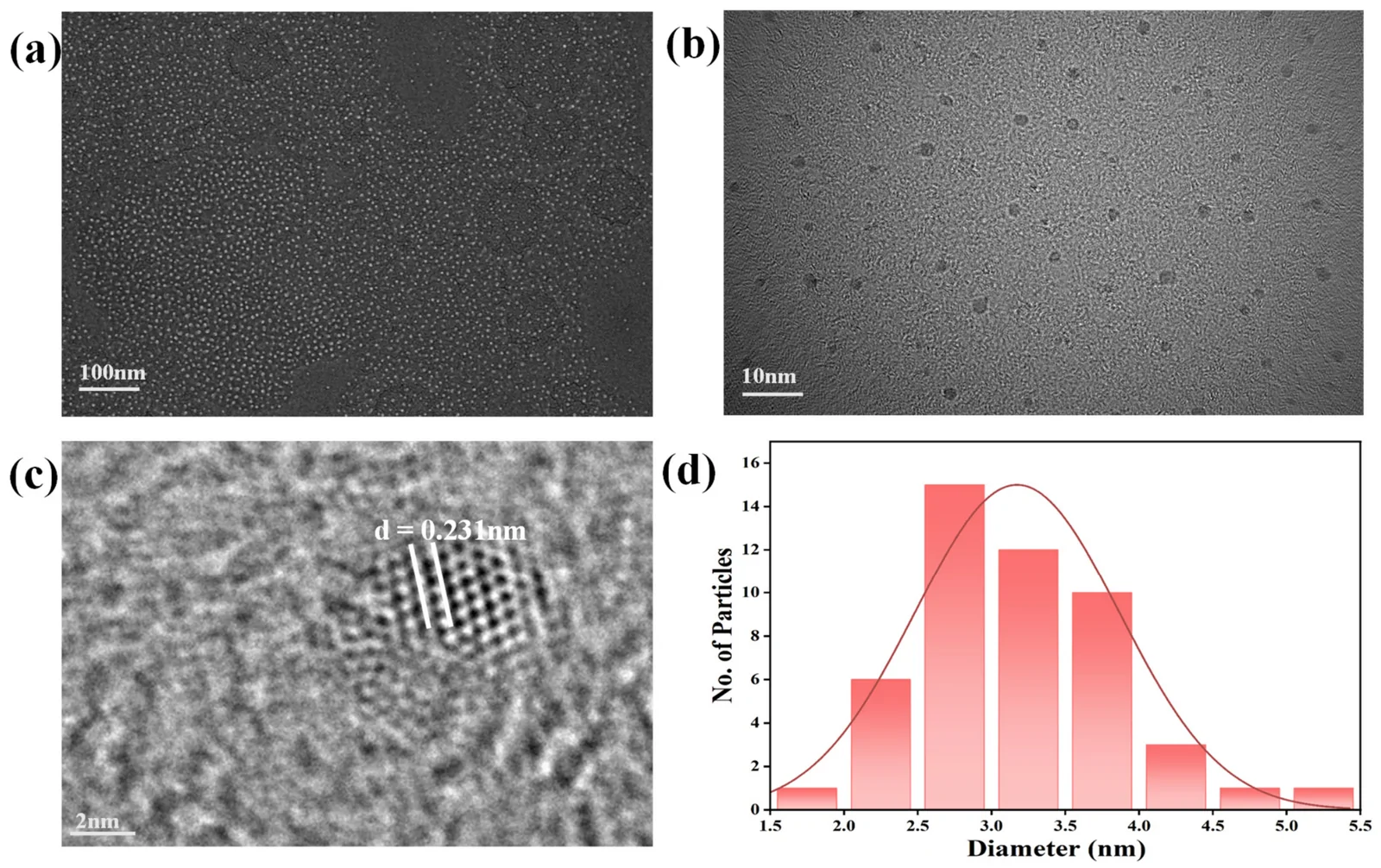
Microstructural and morphological characterization of pristine N-CQDs: (**a**) scanning electron microscopy (SEM) image; (**b**) transmission electron microscopy (TEM) image; (**c**) high-resolution TEM (HRTEM) image (inset); (**d**) particle size distribution histogram.

**Figure 3:**
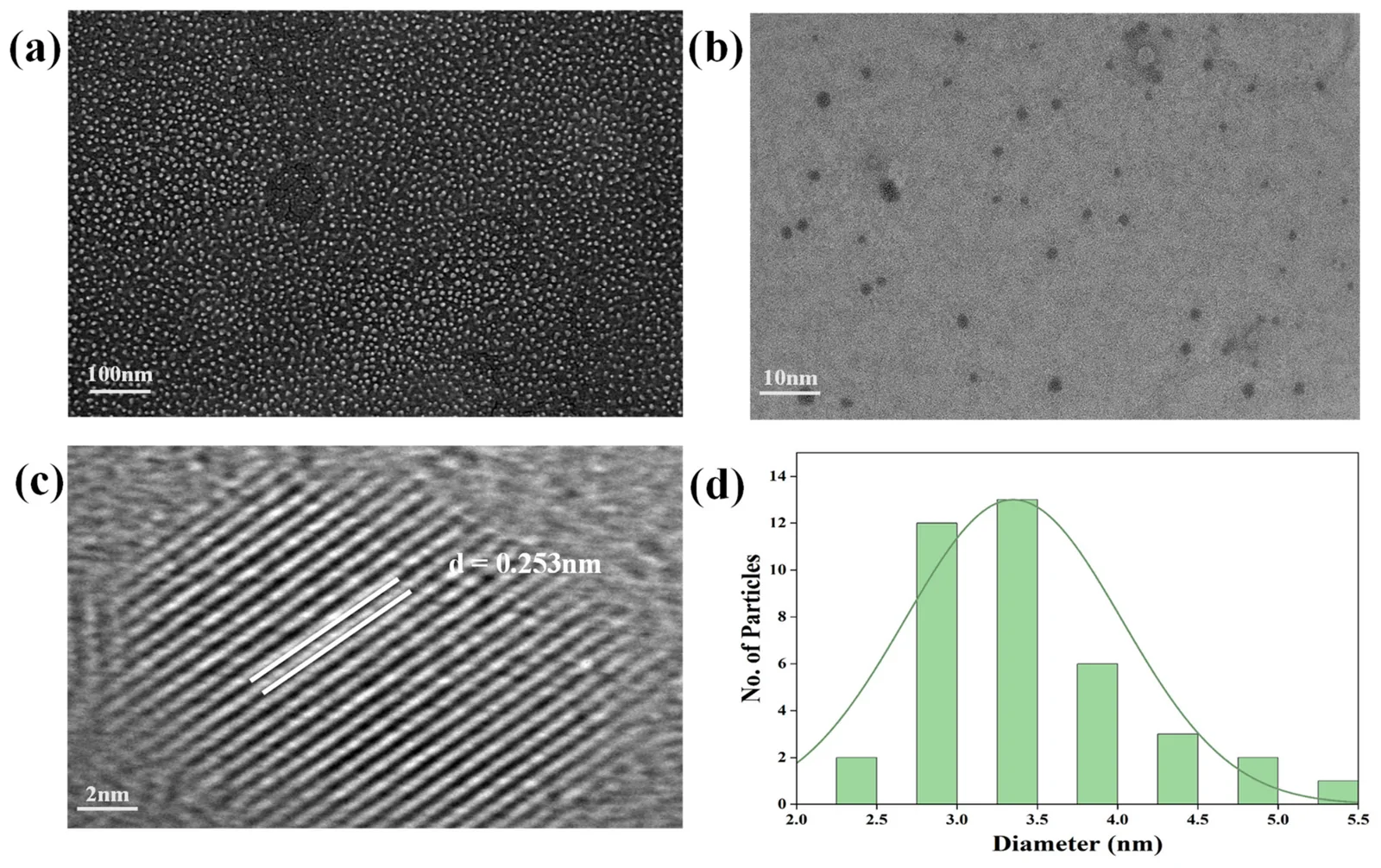
Microstructural and morphological characterization of dual-drug-loaded N-CQDs: (**a**) SEM image; (**b**) TEM image; (**c**) HRTEM image (inset); (**d**) particle size distribution histogram.

The morphological and microstructural features of pristine N-CQDs and drug-loaded N-CQDs were systematically observed using scanning electron microscopy (SEM) and transmission electron microscopy (TEM), and the corresponding results are presented in Figure 2 and Figure 3, respectively [44]. For pristine N-CQDs, the SEM image shown in Figure 2a reveals that the as-synthesized N-CQDs are spherical, uniformly sized, and smooth-surfaced nanoparticles with no obvious aggregation, no large bulk residues, and excellent dispersion, exhibiting a typical homogeneous morphology of high-quality carbon quantum dots. The TEM image in Figure 2b further confirms that the N-CQDs are monodisperse spherical nanoparticles without overlap or agglomeration, indicating the favorable colloidal stability of the prepared samples. The high-resolution TEM (HRTEM) image in the inset of Figure 2c displays distinct and clear lattice fringes, and the measured interplanar spacing is 0.231 nm (Figure 2c), consistent with the in-plane lattice fringe of graphitic carbon, confirming the existence of a partial nanocrystalline structure within the amorphous carbon matrix of the N-CQDs. The particle size distribution histogram in Figure 2d demonstrates that the diameter of pristine N-CQDs ranges from 2.3 nm to 3.7 nm, with an average particle size of 2.76 nm, and the distribution is extremely narrow, reflecting the high controllability and excellent synthesis quality of the hydrothermal preparation process.

For the drug-loaded N-CQDs shown in Figure 3, the SEM image in Figure 3a indicates that the spherical morphology is completely preserved after glucuronic acid functionalization and dual-drug loading, while the surface becomes slightly rougher in comparison with the smooth surface of pristine N-CQDs, which is attributed to the formation of a thin coating layer composed of glucuronic acid and adsorbed drug molecules on the N-CQD surface. Notably, the drug-loaded N-CQDs still maintain excellent dispersion without any agglomeration, verifying the favorable structural and colloidal stability after surface modification and drug loading. The TEM image in Figure 3b shows that the spherical shape of the N-CQDs is well retained, and the particles remain uniformly dispersed, with slightly enhanced contrast at the particle edges, further supporting the presence of surface functionalization and a drug-coating layer. The HRTEM image in the inset of Figure 3c reveals that the characteristic lattice fringes corresponding to the (102) plane are still clearly visible, and clear lattice fringes with an interplanar spacing of 0.253 nm are still visible (Figure 3c)**,** confirming that the internal graphitic core remains intact after functionalization and drug loading. The small increase in apparent spacing relative to the pristine dots is consistent with the outer glucuronic-acid layer and adsorbed drug molecules. The particle size distribution histogram in Figure 3d shows that the diameter of drug-loaded N-CQDs ranges from 2.7 nm to 4.1 nm, with an average particle size of 3.22 nm, representing a uniform increase of approximately 0.46 nm compared with pristine N-CQDs. This moderate increase in size is due to successful glucuronic acid functionalization and the adsorption/encapsulation of piperlongumine and silibinin on the CQD surface. The absence of agglomeration ensures that the particle size distribution remains narrow and uniform, which is favorable for subsequent cellular internalization and biological applications.

#### 3.2.4. X-Ray Photoelectron Spectroscopy (XPS)

**Figure 4:**
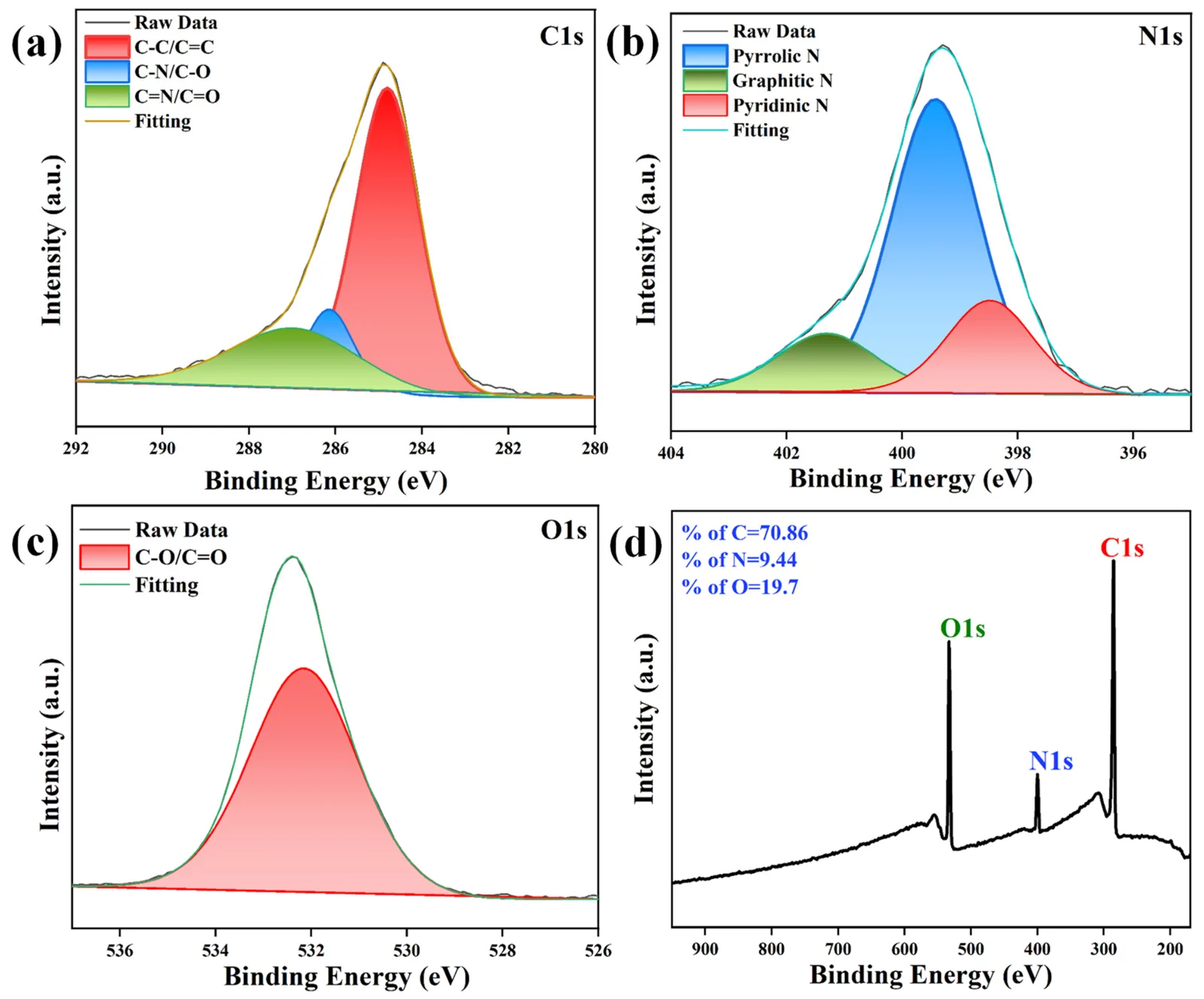
X-ray photoelectron spectroscopy (XPS) analysis of pristine N-CQDs: (**a**) high-resolution C 1s spectrum; (**b**) high-resolution N 1s spectrum; (**c**) high-resolution O 1s spectrum; (**d**) full survey spectrum.

The surface elemental composition and chemical bonding states of the pristine N-CQDs were analyzed using X-ray photoelectron spectroscopy, and the corresponding results are presented in Figure 4 [45]. The full survey XPS spectrum in Figure 4d exhibits three prominent characteristic peaks at binding energies of 284.8 eV, 399.6 eV, and 532.1 eV, which are attributed to C 1s, N 1s, and O 1s, respectively, confirming the successful preparation of nitrogen-doped carbon quantum dots composed of carbon, nitrogen, and oxygen elements. The quantitative elemental analysis reveals that the atomic percentages of carbon, nitrogen, and oxygen in the N-CQDs are 70.86%, 9.44%, and 19.7%, and respectively, and the relatively high oxygen and nitrogen contents provide abundant active sites for subsequent surface functionalization and drug loading. The high-resolution C 1s spectrum in Figure 4a can be deconvoluted into four distinct Gaussian peaks, corresponding to sp^2^ hybridized C=C/C–C bonds at 284.8 eV, sp^3^ hybridized C–C bonds at 285.3 eV, C–O bonds at 286.3 eV, and C=O bonds at 287.5 eV, revealing a complex carbon-containing bonding environment on the CQD surface. The high-resolution N 1s spectrum in Figure 4b is fitted into three component peaks representing different nitrogen doping configurations, including pyridinic N at 399.7 eV, pyrrolic N at 400.2 eV, and graphitic N at 402.2 eV, which collectively contributed to the excellent fluorescence performance and surface reactivity of the N-CQDs. The high-resolution O 1s spectrum in Figure 4c is resolved into two peaks at 531.5 eV and 532.9 eV, which are assigned to C=O and C–O bonds, respectively, further confirming the presence of abundant oxygen-containing functional groups on the CQD surface. This pristine-state composition and bonding profile establishes the abundant carboxyl, hydroxyl, and amine surface groups that serve as the reactive handles for subsequent EDC/NHS coupling with glucuronic acid; the appearance of the amide bands in the FTIR spectrum (Section 3.2.2) directly evidences covalent utilization of these groups for surface modification.

### 3.3. Optical Studies

#### 3.3.1. UV-Vis Absorption Spectroscopy

**Figure 5:**
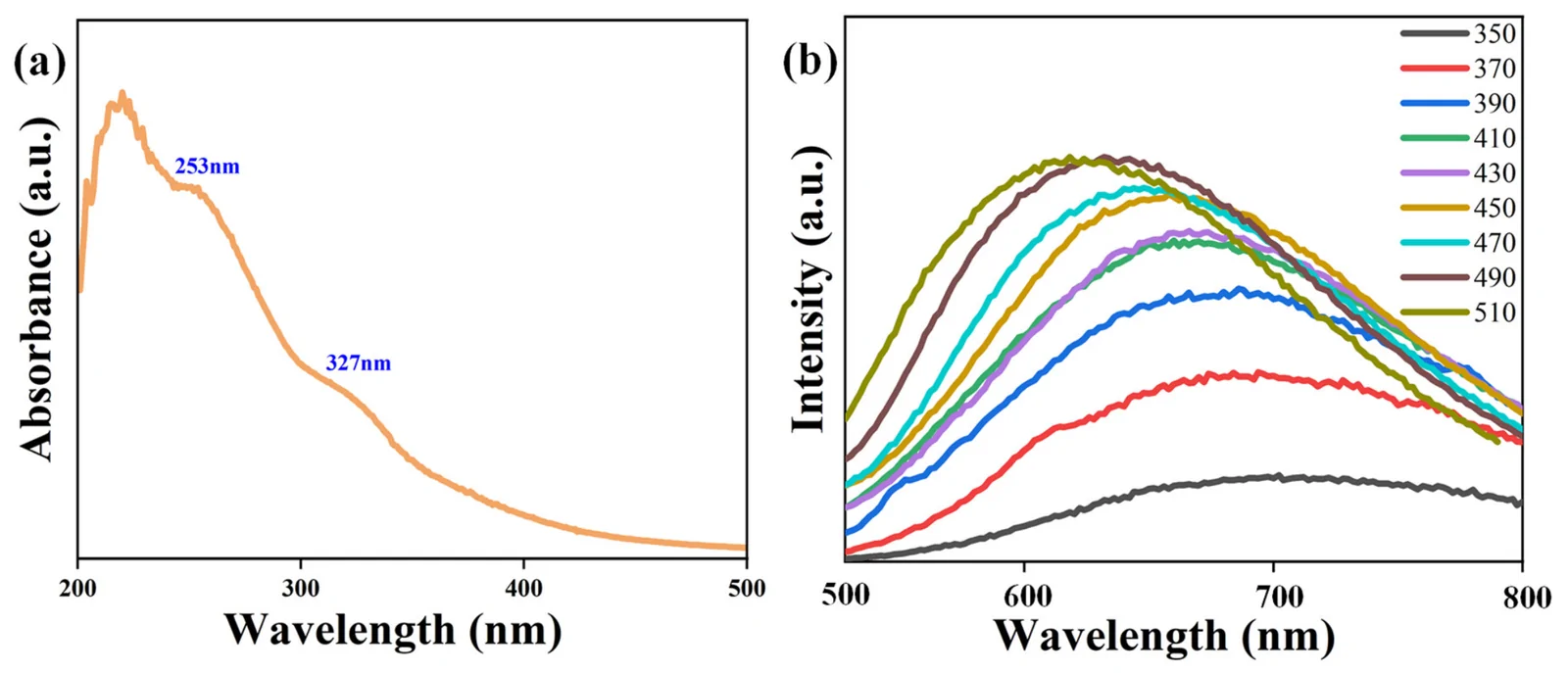
Optical properties of pristine N-CQDs: (**a**) UV-Vis absorption spectrum; (**b**) fluorescence emission spectrum of red-emissive N-CQDs.

The optical absorption properties of the pristine N-CQDs were investigated using UV-Vis. absorption spectroscopy, as displayed in Figure 5a. The UV-Vis. absorption spectrum of the N-CQDs shows two characteristic absorption bands. The strong, sharp absorption peak centered at approximately 215 nm is attributed to the π–π* electronic transition of sp^2^-hybridized conjugated carbon domains in the carbon core of the quantum dots, a characteristic feature of graphitic carbon materials. The relatively broad and weak absorption band at around 272 nm corresponds to the n–π* electronic transition originating from the non-bonding electrons of nitrogen- and oxygen-containing surface functional groups, reflecting the rich surface heteroatom-containing groups of the prepared N-CQDs. After loading of piperlongumine and silibinin, the absorption intensity of the N-CQDs in the range of 250–350 nm is significantly enhanced, which is attributed to the characteristic absorption of the drug molecules and the strong interaction between the drugs and the CQD surface, providing further evidence of successful drug loading.

#### 3.3.2. Photoluminescence (PL) Spectroscopy

**Figure 7:**
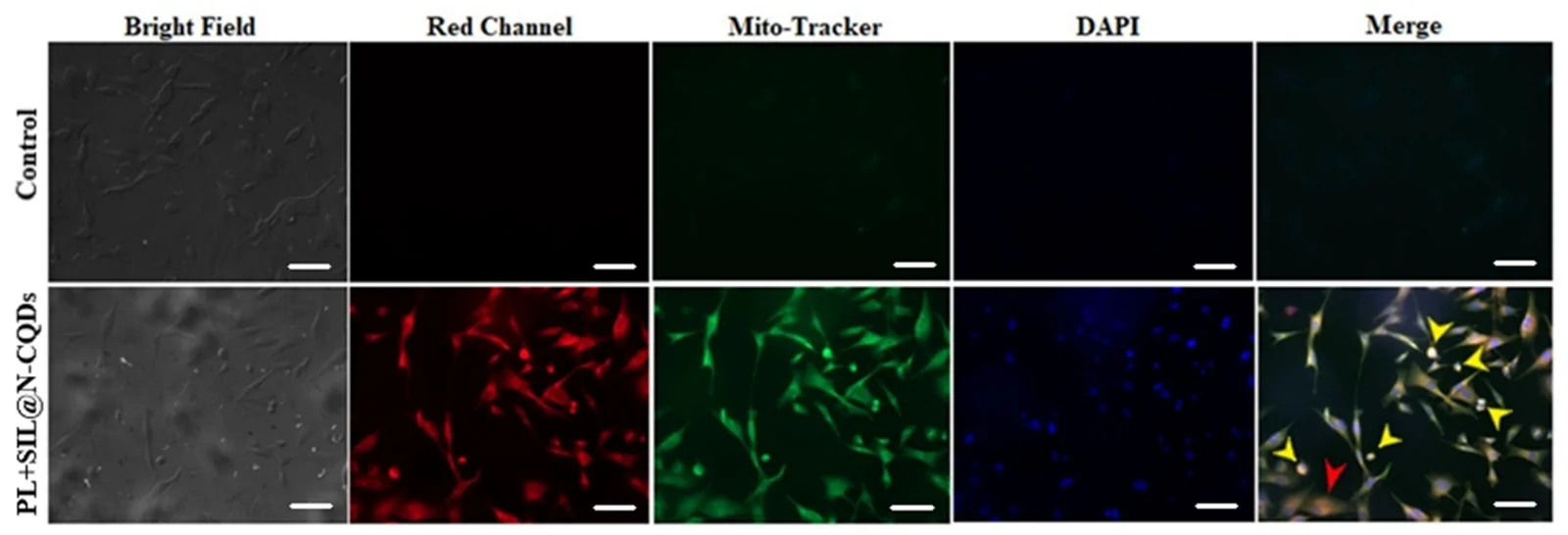
Confocal laser scanning microscopy (CLSM) images of intracellular localization of red-emissive dual-drug-loaded N-CQDs in U87MG glioblastoma cells. Red fluorescence represents N-CQDs; blue fluorescence indicates nuclei stained with DAPI; green fluorescence shows mitochondria stained with Mito-Tracker green. Merged images confirm efficient cellular internalization and mitochondrial targeting (scale bar 10 µm).

The fluorescence properties of the synthesized N-CQDs were characterized using fluorescence spectroscopy, and the results are presented in Figure 5b. The N-CQDs exhibit intense and stable red fluorescence, with a maximum emission peak at 626 nm under an optimal excitation wavelength of 360 nm. The bright red emission feature of the N-CQDs enables high-contrast intracellular fluorescence imaging with minimal interference from cellular autofluorescence, making them ideal for real-time tracking of cellular uptake and subcellular localization in biological applications. After glucuronic acid functionalization and dual-drug loading, the fluorescence intensity of the N-CQDs shows a slight decrease without any obvious shift in the emission peak position, indicating that the surface modification and drug loading processes have little influence on the intrinsic fluorescence emission mechanism of the N-CQDs, and the obtained drug-loaded N-CQDs still retain sufficient fluorescence intensity for bioimaging-guided drug delivery. Both glucuronic-acid functionalization and subsequent dual-drug loading cause only a slight reduction in fluorescence intensity without any appreciable shift in the emission maximum, indicating that the N-doped graphitic emissive core remains intact while surface modification and adsorbed drug molecules introduce modest additional non-radiative decay. The strong, uniform red intracellular fluorescence observed by CLSM after functionalization and loading (Figure 7) confirms that the nanoplatform retains its bright, trackable emission under biological conditions.

### 3.4. Drug Loading and pH-Responsive Release

**Figure 6:**
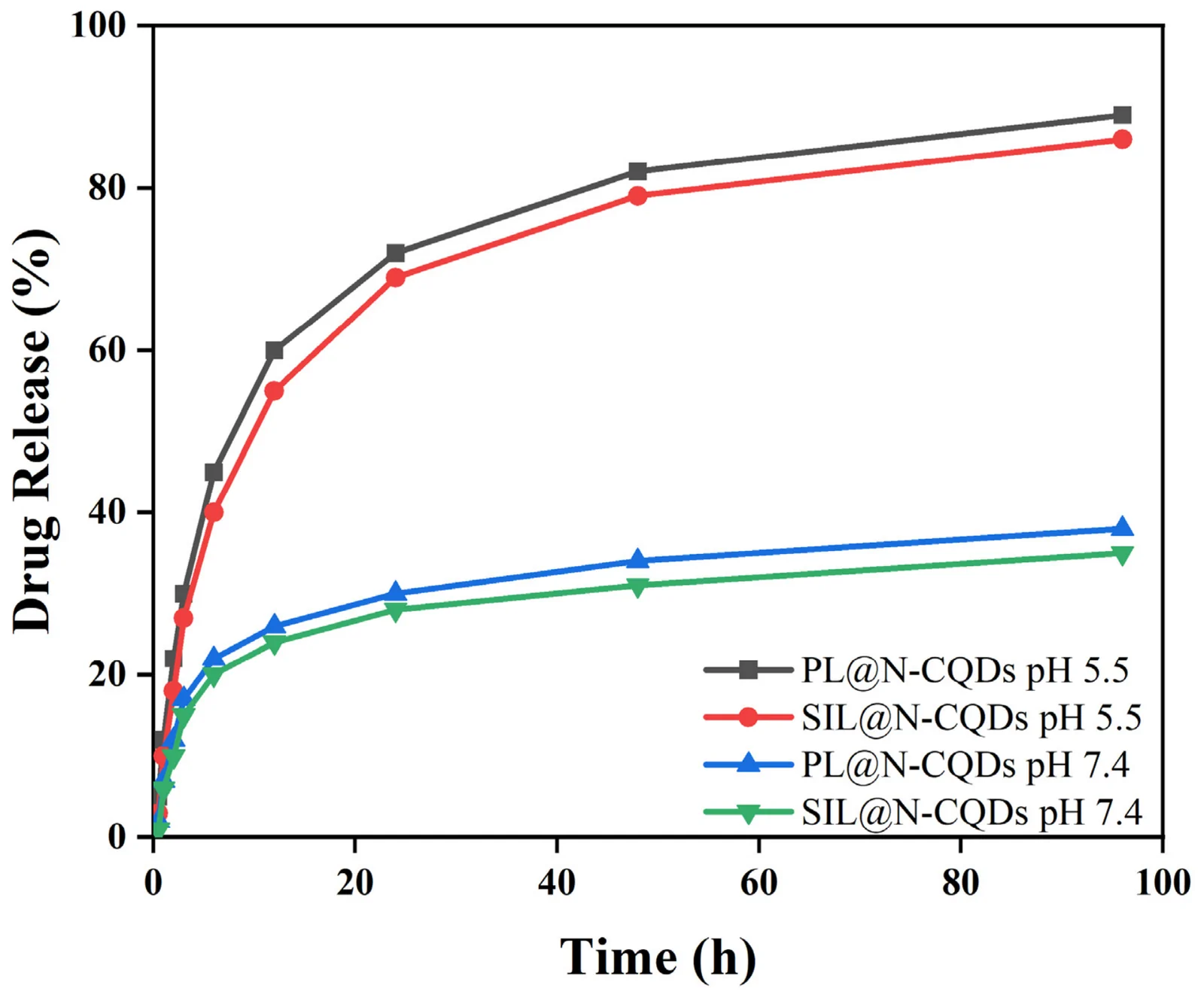
In vitro pH-responsive release profiles of piperlongumine (PL) and silibinin (SIL) from dual-drug-loaded N-CQDs at pH 5.5 (tumor acidic condition) and pH 7.4 (physiological condition) over 96 h.

The drug-loading capacity of the functionalized N-CQDs was quantitatively determined using UV-Vis. spectroscopy, and the results confirmed the successful loading of piperlongumine (PL) and silibinin (SIL) onto the nanocarrier. The drug-loading efficiency for piperlongumine was 18.6%, and that for silibinin was 27.4%, yielding a total dual-drug loading efficiency of 46.0%. Successful loading was quantified by UV-Vis measurement of the free drug remaining in the supernatant (loading efficiency: PL 18.6%, SIL 27.4%, total 46.0%), and the subsequent pH-dependent release of both drugs (Figure 6) further confirms association of the drugs with the carrier. Direct FTIR/XPS characterization of the final drug-loaded product was not performed in this work. Despite the ultrasmall particle size (2.76–3.22 nm) of N-CQDs, efficient dual-drug adsorption and encapsulation are governed by cooperative non-covalent interactions widely validated in the biomass CQD phytotherapeutic delivery literature, including the following: π–π stacking interactions: Conjugated sp^2^ graphitic carbon domains of N-CQDs overlap with aromatic rings within both piperlongumine and silibinin molecular structures, providing dominant adsorption affinity. Hydrogen bonding: Abundant surface hydroxyl, carboxyl and amine groups on GA-functionalized CQDs form intermolecular H-bonds with hydroxyl, carbonyl moieties on PL and SIL. Hydrophobic interactions: The hydrophobic aromatic backbone of PL/SIL partitions onto the partially graphitic hydrophobic carbon core of CQDs, counteracting aqueous solubility of the carrier matrix.

The in vitro release profiles of PL and SIL from the dual-drug-loaded N-CQDs were analyzed over a period of 96 h under simulated physiological conditions at pH 7.4 and tumor acidic microenvironmental conditions at pH 5.5, as illustrated in Figure 6. Under acidic conditions at pH 5.5, the dual-drug-loaded N-CQDs exhibited a rapid initial release phase within the first 24 h, followed by a sustained and controlled release pattern up to 96 h. The cumulative release percentage of piperlongumine reached 89.4% at 96 h, while the cumulative release of silibinin was measured to be 85.1% at the same time point. In sharp contrast, under physiological pH (pH 7.4), both drugs exhibited significantly slower and lower release kinetics, with cumulative release percentages of only 38.2% for PL and 35.6% for SIL at 96 h, respectively.

The remarkable pH-responsive release behavior can be explained by the protonation of surface functional groups on the N-CQDs in an acidic environment, which weakens the electrostatic interactions, hydrogen bonding, and hydrophobic interactions between the drug molecules and the nanocarrier surface. As a result, the loaded drugs are more easily dissociated and released from the quantum dot platform under acidic tumor conditions, while being stably retained under normal physiological pH conditions. This pH-triggered release profile is expected to favor drug liberation at the acidic tumor site while limiting premature release under physiological conditions. However, a direct comparison with free PL + SIL at matched drug doses has not yet been performed, and any incremental benefit of CQD delivery over free drug therefore remains to be established.

The cumulative drug release at pH 7.4 (~38% at 96 h) indicates moderate premature leakage. However, this level of leakage remains therapeutically acceptable because the glucuronic acid-targeting ligand drives preferential accumulation in CD44-overexpressing glioblastoma cells, minimizing off-target exposure. Furthermore, the markedly accelerated release at pH 5.5 (~85–89%) ensures robust drug liberation in the acidic tumor microenvironment, maximizing local therapeutic concentration. Further optimization of surface crosslinking or polymeric shell stabilization may improve physiological retention and reduce premature leakage during systemic circulation. It should be noted that cytotoxicity and apoptosis were evaluated only in U87MG glioblastoma cells; toxicity against non-malignant cells (e.g., normal astrocytes) was not assessed. The reduced release at pH 7.4 provides the mechanistic basis on which tumor-selective liberation is expected, but the actual therapeutic window remains to be verified against normal cells.

### 3.5. Cellular Internalization and Intracellular Localization

The cellular uptake behavior and subcellular distribution of the drug-loaded N-CQDs in U87MG glioblastoma cells were investigated using in vitro confocal laser scanning microscopy (CLSM), as shown in Figure 7. After 4 h of incubation, intense and uniform red fluorescence derived from the N-CQDs was clearly observed within the cytoplasm, indicating that the dual-drug-loaded nanocomposite was efficiently internalized by tumor cells. To further localize the cells, the cells were co-stained with DAPI (blue fluorescence) for nuclear staining and Mito-Tracker Green (green fluorescence) for mitochondrial tracking. The merged CLSM images demonstrated obvious and distinct co-localization of the red fluorescent N-CQDs with the nuclear periphery-region green-fluorescence (mitochondrial localization), as orange fluorescence marked with red arrows, and merged with blue fluorescence (nuclear staining) showing yellow fluorescence, marked with yellow arrows and deformed cell morphology (as compared to that of control elongated cells), confirming the mitochondrial as well as nuclear ability of the prepared nanoplatform. This efficient dual-localization property is highly therapeutically significant, as mitochondria-targeted delivery can effectively amplify intracellular ROS accumulation, leading to caspase-3 activation, which further promotes ROS-mediated apoptosis, while weak nuclear accumulation facilitates direct drug action on genomic DNA, enhancing DNA damage-related cytotoxicity. The combined effects of mitochondrial and nuclear targeting synergize to significantly enhance the overall antitumor activity of the dual-drug-loaded N-CQDs. Although efficient intracellular uptake in U87MG glioblastoma cells was confirmed, this study does not validate the ability to cross the BBB. Future studies will employ an in vitro Transwell BBB model to quantify nanoparticle transcytosis across the brain endothelial barrier.

### 3.6. Anticancer Mechanism: ROS and Caspase-3

**Figure 8:**
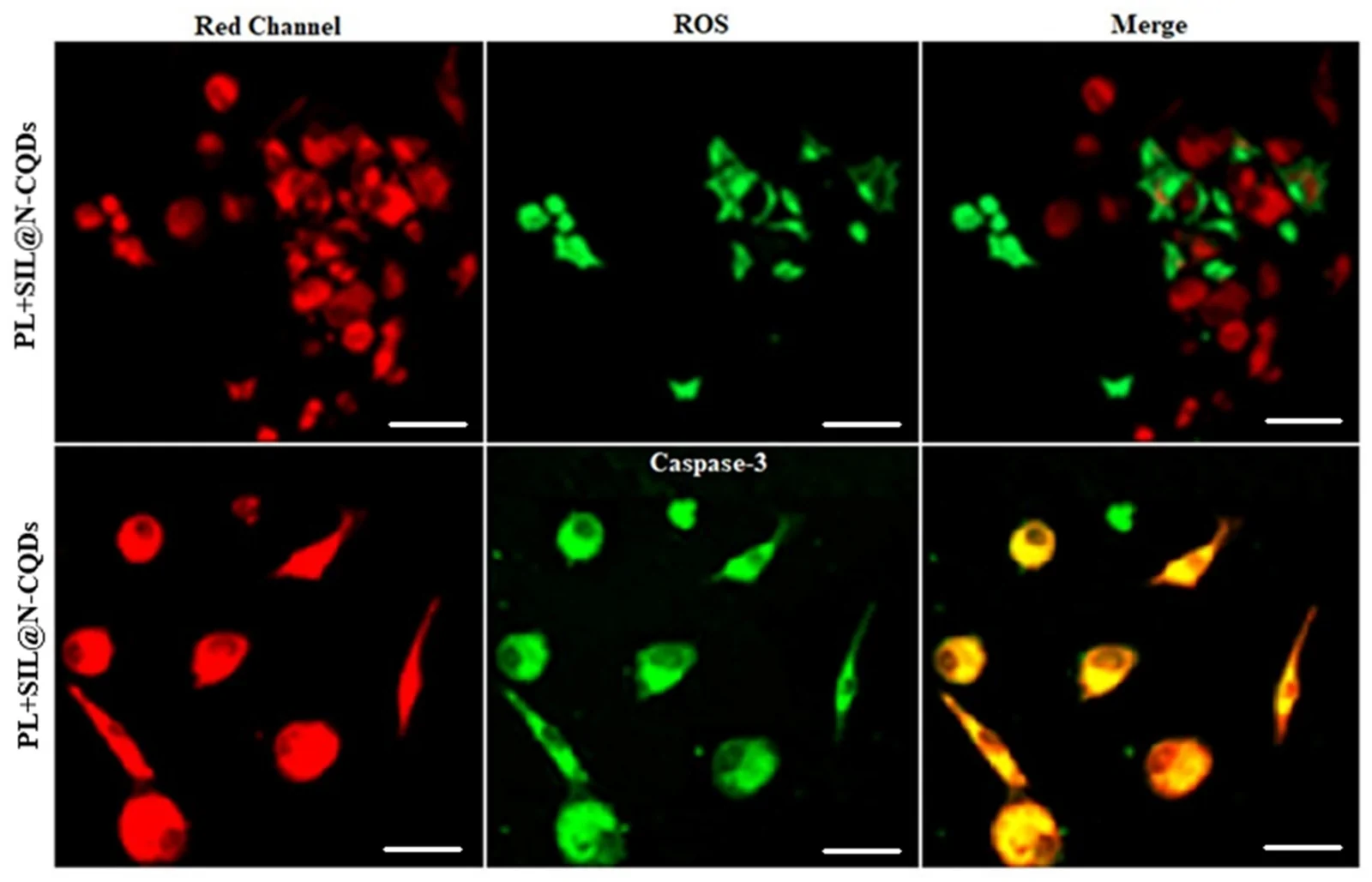
Anticancer mechanism evaluation in U87MG cells treated with dual-drug-loaded N-CQDs: (**left**) intracellular reactive oxygen species (ROS) generation detected by DCFH-DA fluorescence probe; (**right**) caspase-3 activation analysis showing elevated cleaved caspase-3 expression. Enhanced ROS levels and caspase-3 activity confirm the induction of oxidative stress and the caspase-dependent mitochondrial apoptosis pathway (scale bar 20 µm).

To elucidate the underlying molecular mechanism of the enhanced antitumor activity mediated by dual-drug-loaded N-CQDs, intracellular reactive oxygen species (ROS) generation and caspase-3 activation were analyzed, as shown in Figure 8. Compared with the untreated control group, cells treated with dual-drug-loaded N-CQDs showed a significant ~4.8-fold increase in ROS levels (*p* < 0.01), indicating severe oxidative stress and redox imbalance within tumor cells. The excessive presence of ROS induces mitochondrial membrane damage, lipid peroxidation, and DNA strand breakage, which are critical early events that initiate the endogenous mitochondrial apoptosis pathway. In addition, activation of caspase-3, a key executioner protein in the apoptotic signaling cascade, was significantly increased in cells treated with the dual-drug nanosystem, with a nearly 3.6-fold increase in overlapped yellow fluorescence intensity relative to the control group. The marked elevation of cleaved caspase-3 expression confirms the full activation of the caspase-dependent mitochondrial apoptosis pathway. Taken together, the synergistic induction of ROS overproduction and subsequent activation of the caspase-3 cascade constitute the core molecular mechanism by which the dual-drug-loaded N-CQDs exert potent cytotoxicity and induce efficient death of glioblastoma cells.

The synergistic anticancer activity arises from complementary mechanisms: Piperlongumine (PL) selectively induces excessive ROS production, triggering mitochondrial oxidative stress and apoptosis. Silibinin (SIL) acts as a chemosensitizer by suppressing pro-survival signaling pathways, including NF-κB, PI3K/Akt, and STAT3, which are often upregulated in response to PL-induced oxidative stress. This inhibition blocks anti-apoptotic feedback loops, enhances caspase-3 activation, and amplifies apoptotic cell death. Thus, the combined PL + SIL treatment overcomes resistance pathways that limit single-drug efficacy, resulting in significantly higher apoptosis than either drug alone.

### 3.7. Apoptosis/Necrosis Analysis by Flow Cytometry

**Figure 9:**
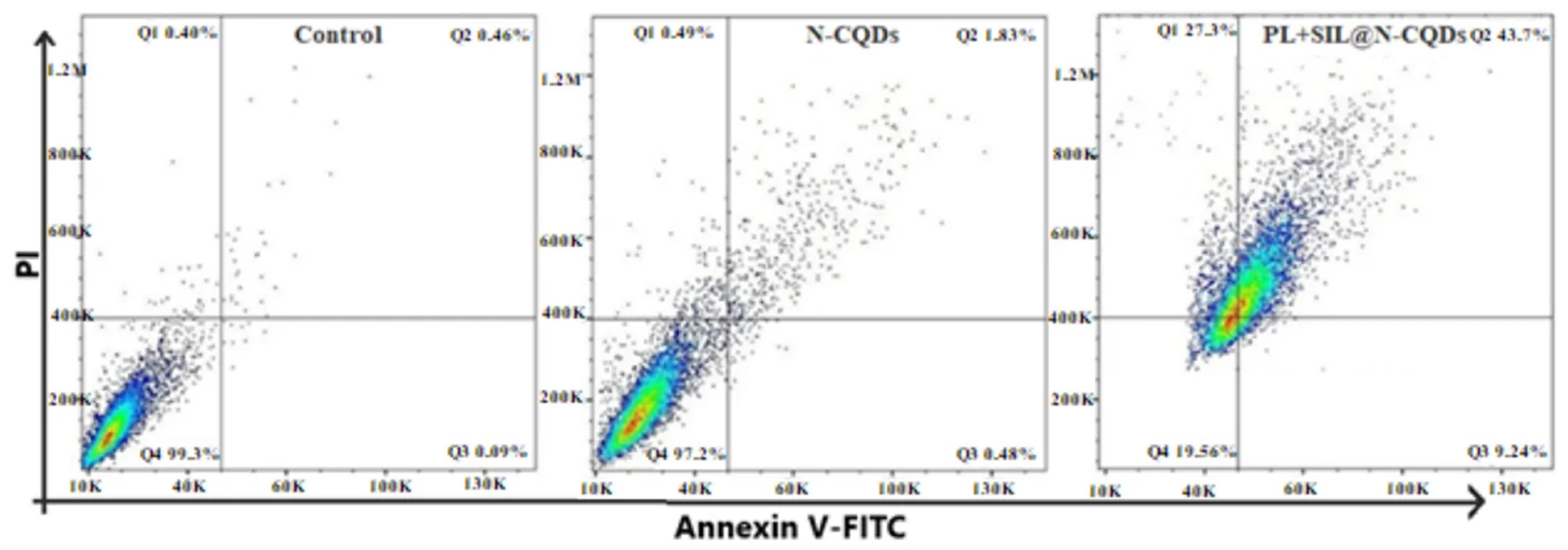
Flow cytometry (FACS) analysis of apoptosis in U87MG glioblastoma cells after treatment (representative of independent experiments; percentages shown are from the representative run).

The CCK-8 assay indicated that blank N-CQDs retained high cell viability over the tested concentration range, confirming good carrier biocompatibility, whereas the drug-loaded formulations reduced U87MG viability in a concentration-dependent manner. The mechanistic assays were carried out at the working concentration selected from this preliminary screen. The pro-apoptotic effects of blank N-CQDs, single-drug-loaded N-CQDs, and dual-drug-loaded N-CQDs against the untreated control group in U87MG glioblastoma cells were quantitatively evaluated using flow cytometry with Annexin V-FITC/PI double staining, and the corresponding quantitative apoptosis rates are displayed in Figure 9. The control group exhibited >99% healthy cells in the Q4 region, whereas cells treated with blank N-CQDs showed a weak apoptosis rate of 2.8% total dead cells, confirming the favorable biocompatibility and negligible cytotoxicity of the unloaded carbon quantum dot nanocarrier. The single-drug-loaded N-CQD groups exhibited improved apoptotic efficiency, with early apoptosis at 38.8% and late apoptosis at 2.8%, leaving only 58.02% live cells, as shown in the Q4 region. Surprisingly, the dual-drug-loaded CQDs formulation induced the strongest apoptotic response, with 9.14% early apoptosis, 43.7% late apoptosis, and 27.3% necrotic cells, leaving only 19.86% live cells and achieving a total apoptosis rate of ~80%, which was significantly higher than that of the drug-free CQDs and single-drug-loaded CQDs groups. The flow-cytometry apoptosis data are shown as a representative experiment; tabulated mean ± SD across independent replicates with full group-wise statistical comparison will be compiled in the extended study. These results clearly confirm the enhanced combined antitumor effect of the piperlongumine–silibinin combination, demonstrating that the dual-drug co-delivery system based on N-CQDs can effectively trigger apoptosis/necrosis in glioblastoma cells and greatly improve chemotherapeutic efficacy. The markedly higher apoptosis rate induced by dual-drug delivery confirms the combined effect of PL-mediated elevation of ROS and SIL-mediated inhibition of survival pathways. It should be noted that the dual-drug group also showed a notable necrotic fraction (27.3%). In a 24-h Annexin V-FITC/PI assay, late-apoptotic cells frequently proceeded to secondary necrosis, so part of this population likely reflects secondary rather than primary necrosis. In addition, the relatively high working concentration selected from preliminary screening may contribute some direct, non-apoptotic cytotoxicity alongside the caspase-3-mediated pathway. Accordingly, the mechanism is best described as predominantly apoptotic with a measurable necrotic component; further dose optimization would be expected to favor the apoptotic arm.

## 4. Limitations

This study has several limitations. All biological evaluations were performed in a single glioblastoma line (U87MG) without a non-malignant control, and active targeting was not directly verified (no blocking assay, CD44-expression or CD44-high/low comparison). No formal Combination-Index synergy analysis, no matched-dose free-drug control, and no tabulated IC_50_ statistics were provided; the flow-cytometry data are representative. In addition, several standard characterizations are missing: DLS/zeta potential, fluorescence quantum yield, FTIR/XPS of the drug-loaded product, long-term stability, hemolysis assays and BBB penetration were not assessed. These points define the scope of the follow-up study.

## 5. Conclusions

This work demonstrates a fully green, hydrothermal fabrication of red-emissive nitrogen-doped carbon quantum dots (N-CQDs) directly from locust biomass, using N,N1-diethyl-p-phenylenediamine as a one-pot nitrogen dopant. Optimization of the dopant-to-biomass ratio yielded narrowly distributed dots (~2.76 nm pristine; ~3.22 nm after loading) with strong red emission (Ex/Em ≈ 490/626 nm) and abundant surface carboxyl, hydroxyl, and amine groups. These reactive groups were used to covalently graft glucuronic acid through EDC/NHS amide coupling equipping the carrier with a receptor-binding ligand candidate and to co-load piperlongumine and silibinin at a total loading of ~46%. The resulting nanoplatform retained the graphitic core and red emission while showing pH-dependent release, with greater drug liberation at pH 5.5 (~85–89%) than at pH 7.4 (~36–38%) over 96 h. In U87MG glioblastoma cells, the platform showed efficient internalization, dual mitochondrial/nuclear subcellular localization, ROS overproduction and caspase-3 activation, producing an enhanced combined apoptotic effect relative to the single-drug controls. The entire route uses renewable biomass, avoids toxic precursors and organic solvents, and aligns with green-nanomedicine principles. As an in vitro proof-of-concept, this work establishes the physicochemical feasibility and initial bioactivity of the platform; direct validation of receptor-mediated targeting, quantitative synergy, a normal-cell therapeutic window, a second glioblastoma line, and in vivo BBB-penetration and efficacy studies remain to be carried out before translational advancement.

## Data Availability

The original contributions presented in this study are included in the article. Further inquiries can be directed to the corresponding author.
